# Squeezing through the microcirculation: survival adaptations of circulating tumour cells to seed metastasis

**DOI:** 10.1038/s41416-020-01176-x

**Published:** 2020-12-01

**Authors:** Julia Perea Paizal, Sam H. Au, Chris Bakal

**Affiliations:** 1 Department of Bioengineering, Imperial College London, London, SW7 2AZ UK; 2 Division of Cancer Biology, Chester Beatty Laboratories, Institute of Cancer Research, 237 Fulham Road, London, SW6 6JB UK; 3 Cancer Research UK Convergence Science Centre, Roderic Hill Building, Imperial College London, London, SW7 2BB UK

**Keywords:** Metastasis, Mechanisms of disease

## Abstract

During metastasis, tumour cells navigating the vascular circulatory system—circulating tumour cells (CTCs)—encounter capillary beds, where they start the process of extravasation. Biomechanical constriction forces exerted by the microcirculation compromise the survival of tumour cells within capillaries, but a proportion of CTCs manage to successfully extravasate and colonise distant sites. Despite the profound importance of this step in the progression of metastatic cancers, the factors about this deadly minority of cells remain elusive. Growing evidence suggests that mechanical forces exerted by the capillaries might induce adaptive mechanisms in CTCs, enhancing their survival and metastatic potency. Advances in microfluidics have enabled a better understanding of the cell-survival capabilities adopted in capillary-mimicking constrictions. In this review, we will highlight adaptations developed by CTCs to endure mechanical constraints in the microvasculature and outline how these mechanical forces might trigger dynamic changes towards a more invasive phenotype. A better understanding of the dynamic mechanisms adopted by CTCs within the microcirculation that ultimately lead to metastasis could open up novel therapeutic avenues.

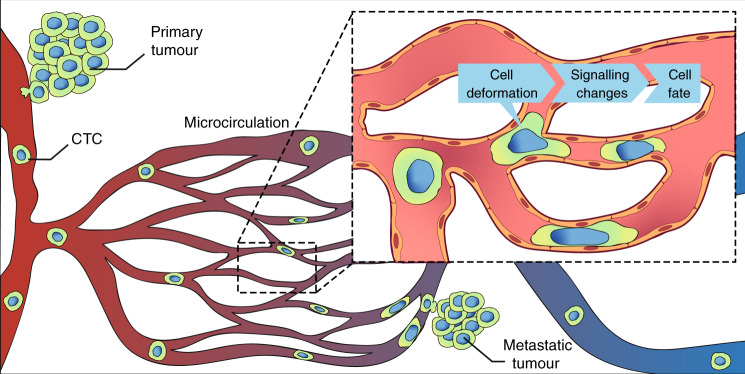

## Background

Metastasis accounts for the majority of cancer-associated deaths, yet it is a highly inefficient process. In animal models, primary tumours shed an estimated 4×10^6^ cells/gram of tumour tissue per day,^1^ but, of this number, fewer than 0.01% manage to successfully extravasate to seed metastases.^2^ Once cancer cells have detached from the primary tumour and entered the bloodstream, they travel as single cells or cell clusters—known as circulating tumour cells (CTCs)—until they undergo arrest in the capillary beds that serve to supply nutrients to, and carry waste away from, the next organ in the circulatory system. CTC arrest can occur as a result of the reduced diameter of the capillary lumen dimensions, although other factors can also influence cell arrest (see below). In the capillary bed, biomechanical forces—predominantly constriction—have been shown to severely deform the cell cytoplasm and nucleus.^3–5^ Although forces exerted on CTCs can result in cell death, capillary constrictions have been estimated to reduce the viability of CTCs entering narrow vessels by as much as 90%.^6^ Kienast et al.^3^ tracked the fate of arrested CTCs in brain capillaries over months using real-time intravital imaging, and discovered that the arrest of CTCs in capillaries is a fundamental step in metastatic progression.^3^ However, how constriction forces that are encountered upon arrest can influence the fates of the remaining cells, and how they might even promote extravasation, has not been fully explored.

Evidence from the past 5 years suggests that CTCs might possess diverse adaptive mechanisms that function in response to constrictive migration to enhance their survival, and also eventually increase their metastatic potency within the microcirculation. One such adaptive mechanism includes the dynamic regulation of cellular stiffness and contractility, which enables the immediate squeezing through narrow constrictions and facilitates extravasation.^7^ Extensive cell deformation can also activate mechanosensitive pathways that might change cell fate over the longer term, such as by engaging cell pro-survival mechanisms^8^ or by inducing epithelial-to-mesenchymal transition (EMT), both of which lead to a more invasive phenotype.^9,10^ Strikingly, some of these adaptations share close analogies with other cell types that typically transit through narrow spaces. For instance, neural crest cells are able to undergo complete or partial EMT during embryonic development to promote migration through micron-size pores,^11,12^ while immune cells can dynamically deform their nuclei^13^ or become activated by mechanical deformation, which has been reported to promote migration in narrow capillaries.^14^

Because there are limited studies focusing on the microcirculation, we will discuss the mechanisms adopted by cancer cells migrating through constricted environments, such as pores with similar dimensions to capillaries. However, while extrapolation of the results of cancer cell migration in constricted microenvironments, such as during tissue invasion, can be useful to understand some of these mechanisms, key differences between migration in these environments and capillaries do not always make such comparisons possible. For instance, while migration of cancer cells in the tumour stroma is thought to be directed by chemoattractant gradients,^15^ the migration of cancer cells in capillaries is controlled by fluid forces.^16,17^ Consequently, the migration of cancer cells through micron-sized pores or capillary constrictions that mimic the extracellular matrix (ECM) can take hours,^18,19^ whereas, when physiological blood flow rates are applied to comparable constrictions, cancer cells are able to overcome them within milliseconds or seconds,^9,20,21^ showing that capillary transit occurs at a faster rate than migration through 3D microenvironments. These substantial differences in the rates of migration between the tumour microenvironment and capillaries might affect the dynamics of acquired adaptations.

**Fig. 1:**
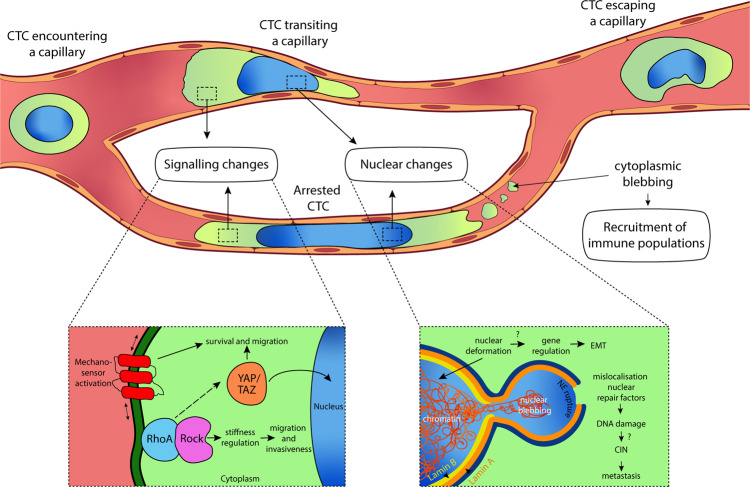
Circulating tumour cell (CTC) encountering a capillary constriction and the possible adaptations that may increase its metastatic potential. When a CTC encounters a capillary bed, two situations can take place: it can get arrested and initiates extravasation, or the CTC can transit through the capillary and escape from it, enabling its migration to a more distant capillary bed. During transit and/or arrest, CTCs undergo intense cell deformation, which can induce changes in the mechanotransduction of signalling pathways, such as RhoA–ROCK and YAP/TAZ,^7,25,47–49^ resulting in an increase of cell invasiveness and survival (see inset “Signalling changes”). Upon activation, YAP/TAZ gets translocated to the nucleus, inducing changes in the transcription activity. The stretching of the cellular membrane favoured by the intense deformation activates mechanosensors that may contribute to cancer progression.^8^ Constriction forces in capillaries also provoke nuclear deformation (see inset “Nuclear changes”), leading to chromatin rearrangement and gene regulation towards epithelial-to-mesenchymal transition (EMT).^9,10,75^ Nuclear deformation can result in the production of nuclear blebs after nuclear lamin rupture, inducing an exchange of nucleo-cytoplasmatic contents and thus, mislocalisation of nuclear repair factors.^10,18^ Consequently, the repair of the nuclear envelope (NE) may be delayed, resulting in an increase in DNA damage. This nuclear damage is one of the possible sources of chromosomal instability (CIN), which has proven to drive metastasis.^80^ Shear forces experienced by CTCs transiting and/or arrested in capillaries can produce cytoplasm blebs, which upon attachment to the capillary wall, attract immune-interacting intermediates that promote extravasation^42^ (see inset “Recruitment of immune populations”). In the event that CTCs manage to squeeze through the capillary preventing arrest, morphological changes are observed in the nucleus and the cytoplasm as a result of severe cell deformation.^9^ Overall, capillary constriction forces may induce long-term effects on cell fate that may contribute to the metastatic potency.

Understanding the behaviour of CTCs in capillaries and how these cells develop adaptation strategies to take on a more invasive phenotype can improve our understanding of metastatic evolution, as the microcirculation is where CTCs usually engage with the endothelium to initiate extravasation^16^—having arrested in the microvasculature, the CTCs extravasate through the thin and permeable vessel walls into the surrounding stroma. In this review, we will describe in vivo and in vitro studies that focus on the effects of biomechanical forces experienced by CTCs in capillary beds during metastasis, and outline how CTCs might adapt to increase their metastatic potential in response to these forces. When encountering a capillary constriction, the cytoplasm is the first cell component that deforms, followed by the nucleus,^22^ due to their different stiffnesses (Fig. 1). We will therefore focus on the deformation experienced by both components and the changes that take place upon encountering and transiting a capillary. As CTC dissemination in the circulation might be an early event in tumour progression,^23^ the development of therapeutics targeting the survival adaptative mechanisms adopted by CTCs in the microvasculature could drastically reduce the potential of CTCs to seed metastasis in distant organs.

## Arrest in capillaries and mechanical deformation

The arrest of CTCs in capillary beds is a complex multifactor step that is essential for metastatic progression. As proposed in Paget’s “seed and soil” hypothesis,^24^ a favourable microenvironment might promote the engagement of CTCs with the endothelium.^16^ Other factors, such as the adhesion potential of CTCs,^17^ the local blood pressure of the organ, its blood flow and the cell deformability, can also influence whether CTCs become arrested.^16,25^ Indeed, Follain et al.^16^ demonstrated in vivo that low blood flow profiles can promote cell arrest without the need for the cells to be physically occluded, suggesting that active adhesion between CTCs and the endothelium is required for a successful extravasation. Other factors, such as the adhesion potential of CTCs^17^ and the mechanical constraints of the capillary,^3,16^ may also determine CTC arrest. For instance, CTCs can be far larger than the diameter of the vessel and/or the architecture of the vessels might be tortuous.^3^ Capillary beds comprise numerous vessel bifurcations, abrupt curvatures and sudden changes in diameter that could facilitate the physical entrapment of CTCs.^16^

CTC clusters, which are composed of cell aggregates, have been reported to have a greater metastatic potential than singlets as well as a shorter half-life in the circulation,^26^ allegedly because they are more likely to undergo arrest owing to their size. Although intense mechanical deformation has been observed in CTC clusters when traversing microvessels,^27^ clusters also possess a survival advantage when compared with singlets, probably due to the cooperation between cells within the cluster, which not only generates hybrid EMT phenotypes,^28^ but can also protect cancer cells against fluid shear stress (FSS) and immune assault by producing a coating shield.^29^ An example of this cooperation can be found in the crosstalk established between cancer cells and neutrophils within the cluster, which has been shown to promote CTC extravasation.^30,31^

### The immediate consequence of capillary forces: nuclear/cytoplasm deformation

Once arrested, CTCs experience mechanical forces similar to those of cells invading 3D tissue or hydrogel,^32^ the effects of which on cell deformation, migration and cell fate, have been extensively studied.^10,18,19,33,34^ Lautscham et al.^32^ observed that the migration velocity of cancer cell lines in confined 3D environments, which can be taken as a measurement of cell invasiveness, was influenced by different factors, including nuclear volume, cell stiffness, cell contractility and adhesiveness. Interestingly, the authors reported that it is the volume of the nucleus, and not the cytoplasm, that limits the ability of cells to migrate through constrictions,^32^ as the nucleus is 5–10 times stiffer than the rest of the cell.^35^ Indeed, in the same study, cell migration was negatively affected by an increase of nuclear stiffness mediated by overexpression of the intermediate filament protein lamin A. By contrast, larger cytoplasmic volumes might actually facilitate migration through narrow pores because they contain a higher number of contractile structures that are capable of generating traction and pulling the cell forward.^32^ In support of this idea, increased adhesion between the cell and the channel—mediated, for example, by coating with the channel with fibronectin and collagen—promoted migration.^32^ Thus, studies of cancer cell invasion suggest that CTC progression through microcapillaries would be inhibited by larger and stiffer nuclei, whereas large cytoplasmic volumes and increased cell–ECM adhesion might facilitate capillary transit.

The viscoelastic properties of cells might also play a role in cell deformation within capillaries, as increased viscoelasticity has been proven to enhance cell migration and invasiveness capacities.^36^ The results from the study of the mechanics of leukocytes, which have a viscoelastic behaviour and demonstrate force relaxation and hysteresis^37^ after applying pulling forces with capillary-sized micropipettes, can be used to better understand the viscoelastic properties of CTCs. Moreover, changes in neutrophil rheology in response to mechanical stimuli have also been reported.^38^ Similarly, the results of experiments with microfluidic channels, in which plastic deformation was observed after repeated cell capillary transit, have revealed the viscoelastic properties of cancer cells.^9,39^ These results suggest that the viscoelastic relaxation time of CTCs, together with their cytoplasmic and nuclear viscosity, might influence the transit and arrest of CTCs in capillaries.

Extensive in vivo studies have also revealed that CTCs transiting microcapillaries might undergo a similar deformation to cells invading 3D tissues. Shape changes in cancer cells transiting capillaries were first observed by intravital imaging using tumour cells expressing green fluorescent protein (GFP) injected in immunocompetent rats.^40^ Using a similar technique, Mook et al.^41^ observed that the arrest of colon cancer cells in the liver was mainly determined by size restrictions between the cancer cell and the capillary.^41^ Advances in the development of dual-fluorescent cell lines enabled the imaging of CTC deformation in capillaries with a higher level of detail. Fibrosarcoma cells transiting through lung and brain microvessels showed extreme elongations of the nucleus and cytoplasm.^4^ Interestingly, different nuclear and cytoplasmic elongation rates could be observed in cancer cells traversing capillaries of the abdomen in mice,^5^ suggesting that deformability of the cytoplasm and nucleus is regulated differently. Additionally, “clasmatosis”, or cytoplasmic fragmentation, a phenomenon that has been confirmed to attract specific populations of immune cells, thereby mediating the efficiency of the metastatic cell seeding,^42^ could be observed in cells transiting narrow capillaries.^5^

### Long-term effects of constrictions on cell fate

Sophisticated in vivo experiments,^16,17,42^ together with the development of microfluidic platforms,^9,19,20,27,43^ have shown how capillary constriction might affect cell fate. Constricted microchannels mimicking capillary constriction forces have been reported to reduce cancer cell viability by 50% upon exit.^20^ Au et al. showed that clusters of CTCs can migrate through narrow capillaries by reorganising into single-file chains, which reduces their hydrodynamic resistance.^27^ Interestingly, after transiting microvascular constrictions, CTCs remained viable and showed no difference in proliferation rates when compared with the control group. However, these studies did not evaluate whether the constrictions imposed by capillaries could confer an advantage on cell migration and metastatic potential. The next few sections will address in-depth how cytoplasmic and nuclear deformation could induce adaptations that drive metastasis.

## Cytoplasmic deformation

While transiting capillary constrictions, the cytoplasm easily deforms, activating different mechanosensors, such as cell-surface channels and mechanotransduction signalling pathways (e.g., involving signalling proteins such as the GTPase RhoA and/or the transcriptional co-activators YAP/TAZ), depicted in Fig. 1. By converting mechanical forces into chemical signals, these proteins can mediate cell-fate decisions in CTCs, thereby enhancing metastasis in response to the cell deformation experienced within capillary beds. Below, we outline some of these mechanotransduction pathways.

### Mechanosensitive signalling pathways that can promote metastasis

One of the main cellular signalling pathways responsible for cytoskeletal regulation in all eukaryotic cells involves the Rho-family GTPase RhoA. Upon activation, RhoA recruits and activates numerous downstream effector proteins, such as Rho-associated protein kinase (ROCK). In turn, ROCK activation stimulates the activity of proteins, such as the myosin II molecular motor, which binds to actin in an ATP-dependent manner, resulting in contraction of actomyosin networks and the upregulation of cell contractility.^44^

Mechanical squeezing of cells in both microfluidic and animal model capillaries, such as in mice and zebrafish, has been reported to increase the activity of RhoA, resulting in the recruitment and activation of ROCK.^25,45^ When CTCs became lodged in capillaries, the subsequent activation of the RhoA–ROCK–myosin II axis upregulated contractility, causing CTCs to change from an elongated to a spherical phenotype. This cellular rounding facilitated the entrapment of CTCs and their subsequent extravasation.^25^ Accordingly, treatment with the ROCK inhibitor fasudil decreased the arrest of individual CTCs and the incidence of tumour metastasis in mice. Moreover, treatment with the myosin II inhibitor blebbistatin over a short period of time (3–6 h) delayed the formation of metastasis and the number of foci.^45^ Finally, myosin II has been reported to be necessary for CTCs to lodge in mouse lung capillaries.^45^ Although in this study myosin II activation was induced by FSS and not by capillary forces, this activation appeared exclusively in cancer cells and not in immune cells, making it an attractive target for future therapeutics.^45^ Thus, RhoA–ROCK–myosin II activity appears to be essential for the morphological adaptations that occur in CTCs during arrest and that result in extravasation during metastasis.

RhoA activation can have immediate effects on the cytoskeleton, on cell–ECM adhesion and, therefore, on cell shape determination, but by activating transcription factors such as YAP/TAZ or SRF, RhoA can also regulate long-term changes in cell fate. For example, Yes-associated protein 1 (YAP1) and its paralogue TAZ are transcriptional co-activators that have previously been found to be regulated by cell density, shape and mechanical stretching.^46^ Lee et al. found that FSS in the lymphatic vasculature (0.05 dyne cm^–2^) promoted the translocation of YAP1/TAZ to the nucleus, which modulated cell motility by activating the ROCK–LIMK–YAP1 signalling axis.^47^ In this study, YAP1 associated with the transcription factor TEAD to promote cancer cell migration, suggesting that the ROCK–LIMK–YAP1 pathway might promote cancer invasiveness and metastasis in the lymphatic system.^47^ In another study, the same FSS were demonstrated to increase cell division by elevating the protein levels of TAZ and promoting its nuclear translocation.^48^ Overall, these results suggest that the FSS experienced by CTCs during their transit and/or arrest in capillaries might activate signalling pathways—triggered by cell deformation—that could enhance the metastatic potency of CTCs.

Nuclear translocation of YAP/TAZ can be directed by forces applied directly to the nucleus^49^ or to the cytoplasm^7^ that might be independent of RhoA activation. Elosegui-Artola et al. showed, using atomic force microscopy (AFM), that force application to the nucleus was sufficient to induce YAP nuclear translocation in the absence of cytoskeletal regulation, due to the transient opening of nuclear pores.^49^ On the other hand, another study combining microfluidic platforms coupled with AFM revealed that the elongated shape adopted by cells migrating through microfluidic channels led to this translocation, resulting in a dynamic regulation of cell stiffness towards a more compliant phenotype that enhanced migration.^7^ Although fluid flow was not included in this study, these results provided direct evidence for a mechanoadaptative mechanism adopted by cancer cells during constricted migration.

### Mechanosensitive channels that can drive cancer progression

Mechanosensitive ion channels can be activated in response to mechanical stimuli,^50^ such as FSS, produced by the frictional forces exerted on CTCs when blood flow passes tangentially over the surface of CTCs.

The cell-surface channel protein pannexin-1 (PANX1), the role of which in metastatic progression has been previously reported,^51,52^ provides an example of how a mechanosensitive channel can regulate CTC survival in the microcirculation. Furlow et al. demonstrated that metastatic breast cancer cells with a PANX1 channel-activating mutation gained a survival advantage due to an increase in the release of ATP, which was modulated via PANX1 when the breast cancer cells became lodged in the microvasculature.^8^ The likely mechanism is that the cellular deformation induced by capillaries forces the stretching of the cellular membrane, which, in turn, opens PANX1 channels. This results in the release of extracellular ATP and subsequent activation of cell-surface purinergic receptors such as P2Y, which are involved in survival signalling during deformation-induced injury.^53^ Consequently, P2Y receptor activation inhibited apoptosis induced by mechanical stress, thereby contributing to metastatic efficiency. Interestingly, therapeutic inhibition of PANX1 channels reduced breast cancer metastasis by increasing cell death within the microvasculature.^8^

Piezo1 and Piezo2 constitute the pore-forming subunits of mechanosensitive ion channels that open in response to mechanical stimuli such as shear stress or membrane stretching.^54^ The activation of Piezo1 channels has proven to sensitise cancer cells to the selective apoptosis inducer TRAIL^55^ in colorectal, prostate and breast cancer cells under circulatory FSS via caspase-dependent apoptosis.^56^ However, Hope et al. discovered that the Piezo1 agonist Yoda1 could trigger TRAIL-mediated apoptosis in static conditions,^55^ suggesting that triggering TRAIL-mediated apoptosis could be used to target different cancer types. Further research into the relevant mechanisms is required, though, as Yoda1 was also found to induce TRAIL sensitisation in non-cancerous human endothelial cells. On the other hand, functional activation of Piezo channels has been reported in cancer cells, in contrast to non-malignant cancer cells. For example, the malignant MCF-7 breast cancer cell line displayed Piezo1 channels that were responsive to an external negative pressure, whereas Piezo1 channels in the benign MCF-10A mammary epithelial cell line did not respond to external stimuli.^57^ In the same study, blocking the mechanosensitive ion channels decreased the motility of MCF-7 cells, but not of the control MCF-10A cell line, suggesting that Piezo1 might play a possible role in tumorigenesis and/or metastasis.^57^ Indeed, migration through confined spaces has been shown to activate Piezo1 as a result of membrane stretching, causing an increase in the intracellular Ca^2+^  concentration.^58^ The authors showed that Piezo1 channels, together with myosin II, promoted the motility of malignant cells in confined spaces and specifically facilitated efficient migration through narrow channels.

Piezo2 channels, which are involved in sensing gentle touch and proprioception, have been shown to be upregulated in the breast cancer cell line MDA-MB-231-BrM2, a subpopulation of MDA-MB-231 cells that specifically metastasises to the brain.^59^ Knockdown of Piezo2 in MDA-MB-231-BrM2 cells resulted in an impaired ability to cross constricted channels of 3 μm, suggesting that Piezo2 channels might also confer an advantage in migration through confined spaces.^59^

Overall, these results suggest that mechanosensors can transduce biomechanical forces imposed by constricted environments into chemical signals that might confer an advantage in metastasis, either by promoting cell migration and extravasation or by protecting CTCs from the extensive deformation and associated apoptosis experienced in capillaries.

### Cytoplasmatic blebbing

CTCs trapped within the microvasculature can undergo cytoplasmic fragmentation, probably as a result of the intense constriction forces experienced upon arrest (Fig. 1). The shedding of blebbed cytoplasmic particles does not compromise nuclear integrity, and the tumour microparticles have been shown to attract subpopulations of myeloid cells after attaching to the lung microvasculature.^42^ Upon phagocytosis of these microparticles, myeloid cells underwent phenotypic changes that increased their extravasation rate, which promoted the development of metastases from the surviving CTCs trapped within the capillaries.^42^ Thus, the release of cytoplasmatic blebs in capillaries, which has been reported to depend not only on cell size but on the mechanical properties of the cell,^60^ could be considered as an adaptation mechanism adopted by CTCs within the microvasculature to favour metastasis.

## Nuclear deformation

In response to capillary-induced constriction, nuclei experience deformations that facilitate cell displacement. Nuclear deformation has been shown to be the main steric hindrance in cells migrating in confined spaces.^32,60,61^ Its regulation can therefore ensure the successful transit through capillary beds, potentially enabling them to travel to an optimal microenvironment where they can get arrested and extravasate (Fig. 1).

### Nuclear displacement in constricted microenvironments

Displacement of the nucleus during migration is brought about by actin retrograde flow, a process in which actin polymerisation occurs from the leading edge of the cell backwards. The ability of actin retrograde flow to move the nucleus away from the leading edge of the cell is mediated through a linker of nucleoskeleton and cytoskeleton (LINC) complexes, which are attached to the nuclear envelope through actin filaments. Disabling LINC complexes has been shown to induce a loss of cellular stiffness comparable with that observed when disabling the nuclear lamina.^62^ Thus, the mechanical coupling between the nucleus and the cytoskeleton through LINC, and consequently with the ECM, mediates the nuclear deformation observed on cells undergoing 3D migration.^63^ In cancer cells migrating in constricted 3D microenvironments, the nucleus experiences a pushing or pulling through actomyosin contractions, which facilitates cell displacement.^22,64^ However, it is likely that a different mechanism of nuclear displacement, which has not yet been investigated in detail, will occur in CTCs transiting the microvasculature.

### Nuclear envelope: lamins

In eukaryotic cells, the nuclear envelope separates the cytoplasm from the nucleus. This barrier, in charge of protecting the genome, comprises three phospholipid bilayer membranes, nuclear membrane proteins, nuclear pore complexes and the nuclear lamina.^65^ The nuclear lamina is a structure composed of a dense network of filaments, which provides support to the nucleus, and its main components are lamin proteins.^66^ Lamin proteins can be grouped as A type (lamins A and C) and B type (lamins B1 and B2), depending on their biochemical function during mitosis.^67^ LMNA encodes lamins A and C, which provide viscous stiffness to the nucleus, whereas B-type lamins confer nuclear elasticity.

Dynamic regulation of the ratio of A- to B-type lamins is a fundamental process during development, during which A lamins have been shown to play an essential role in mechanosensitive differentiation.^22^ During haematopoietic cell maturation, neutrophil maturation requires the downregulation of lamin A/C to achieve a more elastic nucleus suited to migrate through small pores.^22^ Although phosphorylation-mediated turnover of lamin A is thought to be the primary means by which lamin-A levels and, thus, nuclear stiffness, are kept low, cells can also use other mechanisms. For example, dendritic cells use a mechanism based on Arp2/3-mediated actin nucleation to temporarily disrupt the nuclear lamina by weakening lamin A/C, which provides them with sufficient nuclear deformability to pass through small-diameter pores.^34^ Cells with low-lamin A/C expression levels, such as neutrophils, do not depend on this Arp2/3 mechanism to migrate through small pores.^34^

Given the diversity of mechanisms that non-transformed cells can use to modulate lamin levels, is not surprising that cancer cells can alter their lamin levels to adapt to different microenvironments. For instance, cancer cells migrating through small pores (below 6 μm^2^) show altered levels of lamins.^19,33^ As low levels of lamins A and C have been reported to decrease cell stiffness,^18,32,34^ and lower cell stiffness has been correlated with more invasive phenotypes,^64^ it is tempting to speculate that the regulation of CTC stiffness could be an adaptation mechanism of cancer cells to successfully migrate through confined spaces. Indeed, low levels of lamin A/C have been correlated with a worse prognosis and the development of distant metastases in several cancers, such as breast, gastric carcinoma, lymphomas, lung and colon.^68^ Thus, it is likely that only those cancer subpopulations with optimal lamin A/C expression might survive capillary bed entrapment and extravasation. Concurrently, those subpopulations that do not possess this advantage might dynamically decrease their levels of lamin A/C towards a more flexible phenotype when encountering a capillary constriction.

However, reducing lamin levels to decrease stiffness and promote invasion is a double-edged sword for CTCs: although low expression of lamin A/C might favour migration through constricted environments, lamin A/C deficiency can also result in reduced resistance to FSS in CTCs that are transiting the vasculature, leaving the nucleus unprotected during migration and reducing the viability of CTCs.^69^ Harada et al. demonstrated that in order to ensure cell survival during migration through small pores, nuclear stiffness and DNA protection against mechanical forces must be closely balanced, revealing a close interconnection between 3D migration, DNA damage and cancer development.^64^ Consequently, diverse studies have shown that the depletion of lamins increases the rate of rupture of the nuclear envelope,^20,70,71^ resulting in a disruption of cellular functions.^72^ Although transient rupture of the nuclear envelope has been observed in cultured cells during interphase,^70,71,73^ human cancer cells present altered lamin A/C levels and are more likely to experience nuclear envelope rupture than non-cancer cells.^70^

Rupture of the nuclear lamina has been shown to induce the formation of nuclear blebs in cells migrating through constricted environments. During bleb expansion, chromatin can squeeze through the nuclear lamina opening into the bleb,^71,74^ or even detach completely in fragments from the main nucleus.^19,33,70,72^ Eventually, the nuclear envelope collapses and micronuclei are released into the cytoplasm. Lamin A is responsible for restoring nuclear membrane integrity, accumulating at the site of rupture and leaving a “lamin scar” during hours.^19,33^ This nuclear envelope instability due to external mechanical compression is most likely to be a combination of the forces induced by intranuclear pressure due to actin-based nuclear confinement and cytoskeletal forces exerted on the nucleus, as treatment with blebbistatin and cytochalasin has been shown to reduce nuclear envelope rupture rates.^73^ Although nuclear envelope rupture has been reported only in cells migrating through collagen matrices or confined spaces resembling the ECM19, we expect compression capillary forces to be able to induce a similar behaviour on CTCs in terms of the formation of nuclear blebs and the release of cytosolic DNA. The consequences of the rupture of the nuclear envelope and the exchange of nuclear material with the cytoplasm require more investigation to understand whether they might affect the metastatic potency of CTCs. Further research involving microfluidic platforms that mimic capillary constrictions, together with more detailed in vivo studies tracking CTC arrest and extravasation capabilities in relation to their stiffness (i.e., their LMNA expression), is required to elucidate the role that the regulation of cell stiffness has on arrest and metastatic progression.

### Nuclear repair factors

Nuclear envelope rupture, which can occur with and without bleb formation, induces the exchange of nucleocytoplasmic contents^19^ and the mislocalisation of DNA repair factors^75^ (Fig. 1). Currently, it is not completely clear which complexes are required to repair nuclear ruptures. Endosomal sorting complex required for transport III (ESCRT III)^19,33^ and (non-phosphorylated) barrier-to-autointegration factor (BAF)^76^ have both been shown to be relevant for repairing nuclear envelope damage incurred after cells encounter a constriction. Interestingly, ESCRT III normally functions to prevent the occurrence of micronuclei with weak envelopes, but its abnormal accumulation on micronuclei has been seen to intensify DNA damage.^77^ Other repair factors, such as BRCA1, KU80 or 53BP1, which normally diffuse freely into the nucleus,^10^ are significantly depleted in the event of nuclear envelope rupture as they leak into the cytoplasm and their re-entry into the nucleus might take hours.^78^ This might explain the increased levels of DNA damage observed in cells encountering constrictions.^9,19,33^ Additionally, in vitro experiments have reported a delay in mitosis in cancer cells after undergoing capillary constrictions, which can be partially reversed by the co-overexpression of multiple DNA factors or antioxidant inhibitors.^18,79^ DNA damage induced by migration through constrictions that has been demonstrated to be independent of the cell-cycle phase^18^ can also induce genomic instability.^10^

Overall, the mislocalisation of repair factors observed in cells transiting constrictions might promote an increase in DNA damage in cells, which can result in chromosomal instability, one of the hallmarks of cancer that contributes to metastasis.^80^ Further research is required to investigate how capillary constriction forces induce DNA damage and the effects that these DNA alterations might have on genome stability.

### Gene expression regulation towards EMT

Nuclear actin plays a fundamental role in mechanosensing as it provides a viscoelastic mesh inside the nucleus^81^ and is also involved in the stabilisation of chromatin.^82^ External forces are propagated through the actomyosin cytoskeleton to the nuclear lamina, which is connected to the chromatin by chromatin-binding proteins.^83^ Through this connection, cell-extrinsic forces can alter chromatin organisation and mobility,^84^ affecting the mechanical state of chromatin, modulating gene expression and, ultimately, influencing cellular fate.^10,83,85,86^ Within seconds of their application, external forces can deform the nucleus, inducing chromatin stretching and an open chromatin state, thereby facilitating transcription.^87^ However, while undergoing narrow constrictions, chromatin has been shown to suffer local compaction.^88^ Chromatin comprises an estimated 65% of the nuclear volume,^89^ while mobile nucleoplasmic factors such as DNA repair complexes make up the remainder. As local compaction increases the density of chromatin, encountering or transiting narrow constrictions is likely to induce the mislocalisation of mobile nucleoplasmic factors,^89^ thereby delaying DNA repair.^10,18^ Thus, it might be expected, upon transiting and escaping a capillary, or otherwise becoming lodged, that nuclear deformation in CTCs could induce changes in gene regulation by modifying chromatin arrangement,^75^ inducing cell adaptations or even a more proliferative phenotype that promotes invasiveness and metastasis. For instance, migration through constricted environments has been shown to induce changes in the phenotype of cancer cells related to EMT. Levels of the transcription factor GATA,^4^ which drives EMT in cardiogenesis, doubled after successive rounds of migration through micron-sized pores, resulting in a more rapid movement through the pores.^10^ However, it should be considered that the set-up of this experiment, in which cancer cells were exposed to 17 rounds of migration, might not be comparable with the physiological movement of CTCs through capillaries. Cognart et al. reported that the transcription factor Twist2, which has previously been associated with cancer progression,^90,91^ was upregulated in the metastatic line MDA-MB-231 after transiting capillary constrictions.^9^ Additionally, E-cadherin downregulation could be observed upon constriction transit,^9^ although the role of this protein in cancer progression is currently under debate.^92^ These results suggest that capillary constriction forces might be able to induce genomic changes that can promote EMT and cell invasiveness. The specific factors that induce these changes, and their connection to nuclear deformation, need to be further investigated.

## Discussion and conclusions

Cancer cells breaking away from tumours as single cells or cell clusters intravasate, which provides them with a vascular highway around the body. On encountering capillary beds with narrow lumens, CTCs can experience two different situations: a fraction of these cells becomes lodged and manages to successfully extravasate or, alternatively, they squeeze through and migrate to a more distant capillary bed where extravasation might be more convenient. Constriction forces exerted by capillary beds are likely to induce a multitude of effects in CTCs. Although they often compromise cellular integrity, these forces might also confer survival advantages on CTCs that could enhance their metastatic potential. Compression forces might well lead to important fate changes, such as EMT activation, but we do not fully understand yet which factors can activate this regulation within the microvasculature. Furthermore, phenotypes with increased metastatic potential seem to be more resistant to DNA damage within capillaries.^9^ It remains to be determined which mechanisms confer better DNA protection against capillary constrictions, and whether the DNA damage that is induced might be directly connected to an increase in genetic instability that could enhance the metastatic potency. Thus, further research characterising cell behaviour before and after capillary transit is required, including dynamic cell studies assessing gene expression and EMT activation towards more metastatic phenotypes.

As few studies have specifically focused on the role of constriction forces in capillary vessels, we believe that future research should combine microfluidic technology and organ-on-chip systems to recapitulate the capillary microenvironment. Microfluidic platforms, which can mimic capillary constriction geometries while implementing physiological fluid flows, could improve our ability to understand the diverse role of capillaries in the metastatic cascade.

Furthermore, cellular heterogeneity should be taken into consideration more in future investigation. Most in vitro studies focusing on CTC transit or cell migration in constricted environments use cell lines that do not fully reflect or recapitulate the heterogeneity found in CTCs,^93^ which can exist as single cells or clusters in the circulation.^26^ As it is expected that tumour heterogeneity and organotropism might affect the behaviour of tumour cells and their possible adaptations within capillary beds,^94^ we believe that high-throughput systems that enable the study of large numbers of cells, combined with mRNA, cell signalling and proteome quantification, will help to include and characterise this heterogeneity, contributing to understanding how CTCs with different phenotypes might react in constricted microenvironments.

Overall, investigating how CTCs react to migration through capillaries, and how the constriction forces experienced might impact their survival and invasiveness, can help to understand the role that the microcirculation plays in the metastatic cascade. This knowledge can be used to target metastasis more efficiently by designing therapeutics that inhibit the possible adaptation mechanisms adopted by CTCs in the capillary bed. As CTCs are expected to spend a matter of seconds freely diffusing into the circulation, but hours to days arrested in capillary beds until extravasation takes place, we believe that targeting possible adaptations of CTCs within capillaries is a promising approach to blocking metastatic progression.

## Data Availability

Not applicable.

## References

[1] Butler TP, Gullino PM (1975). Quantitation of cell shedding into efferent blood of mammary adenocarcinoma. Cancer Res..

[2] Yoshida K, Fujikawa T, Tanabe A, Sakurai K (1993). Quantitative analysis of distribution and fate of human lung cancer emboli labeled with 125I-5-iodo-2′-deoxyuridine in nude mice. Surg. Today.

[3] Kienast, Y., Baumgarten, L. Von, Fuhrmann, M., Klinkert, W. E. F., Goldbrunner, R., Herms, J. et al. Real-time imaging reveals the single steps of brain metastasis formation. *Nat. Med.* **16**, 116–122 (2010). 10.1038/nm.2072 20023634

[4] Yamamoto N, Jiang P, Yang M, Xu M, Yamauchi K, Tsuchiya H (2004). Cellular dynamics visualized in live cells in vitro and in vivo by differential dual-color nuclear-cytoplasmic fluorescent-protein expression. Cancer Res..

[5] Yamauchi, K., Yang, M., Jiang, P., Yamamoto, N., Xu, M., Amoh, Y. et al. Real-time in vivo dual-color imaging of intracapillary cancer cell and nucleus deformation and migration. *Cancer Res.* **65**, 4246–4253 (2005). 10.1158/0008-5472.CAN-05-0069 15899816

[6] Weiss L, Nannmark U, Johansson BR, Bagge U (1992). Lethal deformation of cancer cells in the microcirculation: a potential rate regulator of hematogenous metastasis. Int. J. Cancer.

[7] Rianna C, Radmacher M, Kumar S (2020). Direct evidence that tumor cells soften when navigating confined spaces. Mol. Biol. Cell..

[8] Furlow PW, Zhang S, Soong TD, Halberg N, Goodarzi H, Mangrum C (2015). Mechanosensitive pannexin-1 channels mediate microvascular metastatic cell survival. Nat. Cell Biol..

[9] Cognart HA, Viovy JL, Villard C (2020). Fluid shear stress coupled with narrow constrictions induce cell type-dependent morphological and molecular changes in SK-BR-3 and MDA-MB-231 cells. Sci. Rep..

[10] Irianto, J., Xia, Y., Pfeifer, C. R., Athirasala, A., Ji, J., Alvey, C. et al. DNA damage follows repair factor depletion and portends genome variation in cancer cells after pore migration. *Curr. Biol.* **27**, 210–223 (2018). 10.1016/j.cub.2016.11.049 PMC5262636 27989676

[11] Theveneau E, Mayor R (2012). Neural crest delamination and migration: from epithelium-to-mesenchyme transition to collective cell migration. Dev. Biol..

[12] Gallik KL, Ahsan K, Treffy RW, Nacke LM, Green-Saxena A, Rocha M (2017). Neural crest and cancer: divergent travelers on similar paths. Mech. Dev..

[13] Barzilai S, Yadav SK, Morrell S, Zemel A, Nourshargh S, Alon R (2017). Leukocytes breach endothelial barriers by insertion of nuclear lobes and disassembly of endothelial actin filaments. CellReports.

[14] Yap B, Kamm RD (2005). Mechanical deformation of neutrophils into narrow channels induces pseudopod projection and changes in biomechanical properties. J. Appl Physiol..

[15] Roussos ET, Condeelis JS, Patsialou A (2011). Chemotaxis in cancer. Nat. Rev. Cancer.

[16] Follain G, Osmani N, Azevedo S, Allio G, Mercier L, Karreman MA (2018). Hemodynamic forces tune the arrest, adhesion and extravasation of circulating tumor cells. Dev. Cell..

[17] Osmani N, Follain G, García León MJ, Lefebvre O, Busnelli I, Larnicol A (2019). Metastatic tumor cells exploit their adhesion repertoire to counteract shear forces during intravascular arrest. Cell Rep..

[18] Pfeifer CR, Xia Y, Zhu K, Liu D, Irianto J, Weaver VM (2018). Constricted migration increases DNA damage and independently represses cell cycle. Mol. Biol. Cell..

[19] Denais CM, Gilbert RM, Isermann P, McGregor AL, Te Lindert M, Weigelin B (2016). Nuclear envelope rupture and repair during cancer cell migration. Science.

[20] Nath B, Raza A, Sethi V, Dalal A, Ghosh SS, Biswas G (2018). Understanding flow dynamics, viability and metastatic potency of cervical cancer (HeLa) cells through constricted microchannel. Sci. Rep..

[21] Lange JR, Steinwachs J, Kolb T, Lautscham LA, Harder I, Whyte G (2015). Microconstriction arrays for high-throughput quantitative measurements of cell mechanical properties. Biophys. J..

[22] Swift, J., Ivanovska, I. L., Buxboim, A., Harada, T., Dingal, P. C. D. P., Pinter, J. et al. Nuclear lamin-A scales with tissue stiffness and enhances matrix-directed differentiation. *Science* **341**, 1240104 (2013). 10.1126/science.1240104 PMC3976548 23990565

[23] Friberg S, Nyström A (2015). Cancer metastases: early dissemination and late recurrences. Cancer Growth Metastasis..

[24] Paget S (1889). The distribution of secondary growth in cancer of the breast. Lancet.

[25] Huang X, Yang Y, Zhao Y, Cao D, Ai X, Zeng A (2018). RhoA-stimulated intra-capillary morphology switch facilitates the arrest of individual circulating tumor cells. Int. J. Cancer.

[26] Aceto N, Bardia A, Miyamoto DT, Donaldson MC, Wittner BS, Spencer JA (2014). Circulating tumor cell clusters are oligoclonal precursors of breast cancer metastasis. Cell.

[27] Au SH, Storey BD, Moore JC, Tang Q, Chen YL, Javaid S (2016). Clusters of circulating tumor cells traverse capillary-sized vessels. Proc. Natl Acad. Sci. USA.

[28] Genna A, Vanwynsberghe AM, Villard AV, Pottier C, Ancel J, Polette M (2020). Emt-associated heterogeneity in circulating tumor cells: Sticky friends on the road to metastasis. Cancers.

[29] Giuliano M, Shaikh A, Lo HC, Arpino G, De Placido S, Zhang XH (2018). Perspective on circulating tumor cell clusters: Why it takes a village to metastasize. Cancer Res..

[30] Chen MB, Hajal C, Benjamin DC, Yu C, Azizgolshani H, Hynes RO (2018). Inflamed neutrophils sequestered at entrapped tumor cells via chemotactic confinement promote tumor cell extravasation. Proc. Natl Acad. Sci. USA.

[31] Szczerba BM, Castro-Giner F, Vetter M, Krol I, Gkountela S, Landin J (2019). Neutrophils escort circulating tumour cells to enable cell cycle progression. Nature.

[32] Lautscham LA, Kämmerer C, Lange JR, Kolb T, Mark C, Schilling A (2015). Migration in confined 3D environments is determined by a combination of adhesiveness, nuclear volume, contractility, and cell stiffness. Biophys. J..

[33] Raab M, Gentili M, De Belly H, Thiam HR, Vargas P, Jimenez AJ (2016). ESCRT III repairs nuclear envelope ruptures during cell migration to limit DNA damage and cell death. Science.

[34] Thiam HR, Vargas P, Carpi N, Crespo CL, Raab M, Terriac E (2016). Perinuclear Arp2/3-driven actin polymerization enables nuclear deformation to facilitate cell migration through complex environments. Nat. Commun..

[35] Lammerding J (2011). Mechanics of the nucleus. Compr. Physiol..

[36] de Sousa JS, Freire RS, Sousa FD, Radmacher M, Silva AFB, Ramos MV (2020). Double power-law viscoelastic relaxation of living cells encodes motility trends. Sci. Rep..

[37] Xu G, Shao JY (2008). Human neutrophil surface protrusion under a point load: Location independence and viscoelasticity. Am. J. Physiol. - Cell Physiol..

[38] Pai A, Sundd P, Tees DFJ (2008). In situ microrheological determination of neutrophil stiffening following adhesion in a model capillary. Ann. Biomed. Eng..

[39] Kamyabi N, Khan ZS, Vanapalli SA (2017). Flow-induced transport of tumor cells in a microfluidic capillary network: role of friction and repeated deformation. Cell Mol. Bioeng..

[40] Farina KL, Wyckoff JB, Rivera J, Lee H, Segall JE, Condeelis JS (1998). Cell motility of tumor cells visualized in living intact primary tumors using green fluorescent protein. Cancer Res..

[41] Mook O (2003). Visualization of early events in tumor formation of eGFP-transfected rat colon cancer cells in liver. Hepatology.

[42] Headley MB, Bins A, Nip A, Roberts EW, Looney MR, Gerard A (2016). Visualization of immediate immune responses to pioneer metastatic cells in the lung. Nature.

[43] Chen MB, Whisler JA, Fröse J, Yu C, Shin Y, Kamm RD (2017). On-chip human microvasculature assay for visualization and quantification of tumor cell extravasation dynamics. Nat. Protoc..

[44] Kimura K, Ito M, Amano M, Chihara K, Fukata Y, Nakafuku M (1996). Regulation of myosin phosphatase by Rho and Rho-associated kinase (Rho- kinase). Science.

[45] Moose DL, Krog BL, Kim TH, Zhao L, Williams-Perez S, Burke G (2020). Cancer cells resist mechanical destruction in circulation via RhoA/actomyosin-dependent mechano-adaptation. Cell Rep..

[46] Dupont S, Morsut L, Aragona M, Enzo E, Giulitti S, Cordenonsi M (2011). Role of YAP/TAZ in mechanotransduction. Nature.

[47] Lee HJ, Diaz MF, Price KM, Ozuna JA, Zhang S, Sevick-Muraca EM (2017). Fluid shear stress activates YAP1 to promote cancer cell motility. Nat. Commun..

[48] Lee HJ, Ewere A, Diaz MF, Wenzel PL (2018). TAZ responds to fluid shear stress to regulate the cell cycle. Cell Cycle.

[49] Elosegui-Artola A, Andreu I, Beedle AEM, Lezamiz A, Uroz M, Kosmalska AJ (2017). Force triggers YAP nuclear entry by regulating transport across nuclear pores. Cell.

[50] Martial S (2016). Involvement of ion channels and transporters in carcinoma angiogenesis and metastasis. Am. J. Physiol. Physiol..

[51] Penuela S, Gyeniss L, Ablack A, Churko JM, Berger AC, Litchfield DW (2012). Loss of pannexin 1 attenuates melanoma progression by reversion to a melanocytic phenotype. J. Biol. Chem..

[52] Shi G, Liu C, Yang Y, Song L, Liu X, Wang C (2019). Panx1 promotes invasion-metastasis cascade in hepatocellular carcinoma. J. Cancer.

[53] Belete, H. A., Hubmayr, R. D., Wang, S. & Singh, R. D. The role of purinergic signaling on deformation induced injury and repair responses of alveolar epithelial cells. *PLoS ONE* **6**, e27469 (2011). 10.1371/journal.pone.0027469 PMC3210789 22087324

[54] Coste B, Mathur J, Schmidt M, Earley TJ, Ranade S, Petrus MJ (2010). Piezo1 and Piezo2 are essential components of distinct mechanically activated cation channels. Science.

[55] Hope JM, Lopez-Cavestany M, Wang W, Reinhart-King CA, King MR (2019). Activation of Piezo1 sensitizes cells to TRAIL-mediated apoptosis through mitochondrial outer membrane permeability. Cell Death Dis..

[56] Mitchell MJ, King MR (2013). Fluid shear stress sensitizes cancer cells to receptor-mediated apoptosis via trimeric death receptors. N. J. Phys..

[57] Li C, Rezania S, Kammerer S, Sokolowski A, Devaney T, Gorischek A (2015). Piezo1 forms mechanosensitive ion channels in the human MCF-7 breast cancer cell line. Sci. Rep..

[58] Hung W-C, Yang JR, Yankaskas CL, Wong BS, Wu P-H, Pardo-Pastor C (2016). Confinement sensing and signal optimization via Piezo1/PKA and myosin II pathways. Cell Rep..

[59] Pardo-Pastor C, Rubio-Moscardo F, Vogel-González M, Serra SA, Afthinos A, Mrkonjic S (2018). Piezo2 channel regulates RhoA and actin cytoskeleton to promote cell mechanobiological responses. Proc. Natl Acad. Sci. USA.

[60] Kamyabi, N. & Vanapalli, S. A. Microfluidic cell fragmentation for mechanical phenotyping of cancer cells. *Biomicrofluidics* **10**, 021102 (2016). 10.1063/1.4944057 PMC4798995 27042246

[61] Xia Y, Wan Y, Hao S, Nisic M, Harouaka RA, Chen Y (2018). Nucleus of circulating tumor cell determines its translocation through biomimetic microconstrictions and its physical enrichment by microfiltration. Small.

[62] Gomes ER, Jani S, Gundersen GG (2005). Nuclear movement regulated by Cdc42, MRCK, myosin, and actin flow establishes MTOC polarization in migrating cells. Cell.

[63] Stewart-Hutchinson PJ, Hale CM, Wirtz D, Hodzic D (2008). Structural requirements for the assembly of LINC complexes and their function in cellular mechanical stiffness. Exp. Cell Res..

[64] Harada T, Swift J, Irianto J, Shin JW, Spinler KR, Athirasala A (2014). Nuclear lamin stiffness is a barrier to 3D migration, but softness can limit survival. J. Cell Biol..

[65] D’Angelo MA, Hetzer MW (2006). The role of the nuclear envelope in cellular organization. Cell Mol. Life Sci..

[66] Gruenbaum Y, Margalit A, Goldman RD, Shumaker DK, Wilson KL (2005). The nuclear lamina comes of age. Nat. Rev. Mol. Cell Biol..

[67] Dahl KN, Kahn SM, Wilson KL, Discher DE (2004). The nuclear envelope lamina network has elasticity and a compressibility limit suggestive of a molecular shock absorber. J. Cell Sci..

[68] Alhudiri IM, Nolan CC, Ellis IO, Elzagheid A, Rakha EA, Green AR (2019). Expression of lamin A/C in early-stage breast cancer and its prognostic value. Breast Cancer Res. Treat..

[69] Mitchell MJ, Denais C, Chan MF, Wang Z, Lammerding J, King MR (2015). Lamin A/C deficiency reduces circulating tumor cell resistance to fluid shear stress. Am. J. Physiol. - Cell Physiol..

[70] Vargas JD, Hatch EM, Anderson DJ, Hetzer MW (2012). Transient nuclear envelope rupturing during interphase in human cancer cells. Nucleus.

[71] De vos WH, Houben F, Kamps M, Malhas A, Verheyen F, Cox J (2011). Repetitive disruptions of the nuclear envelope invoke temporary loss of cellular compartmentalization in laminopathies. Hum. Mol. Genet..

[72] Robijns J, Molenberghs F, Sieprath T, Corne TDJ, Verschuuren M, De, Vos WH (2016). In silico synchronization reveals regulators of nuclear ruptures in lamin A/C deficient model cells. Sci. Rep..

[73] Hatch EM, Hetzer MW (2016). Nuclear envelope rupture is induced by actin-based nucleus confinement. J. Cell Biol..

[74] Denais C, Lammerding J, Exp A, Biol M, Denais C, Lammerding J (2015). Nuclear mechanics in cancer. Adv. Exp. Med. Biol..

[75] Irianto J, Pfeifer CR, Bennett RR, Xia Y, Ivanovska IL, Liu AJ (2016). Nuclear constriction segregates mobile nuclear proteins away from chromatin. Mol. Biol. Cell..

[76] Halfmann CT, Sears RM, Katiyar A, Busselman BW, Aman LK, Zhang Q (2019). Repair of nuclear ruptures requires barrier-to-autointegration factor. J. Cell Biol..

[77] Willan J, Cleasby AJ, Flores-Rodriguez N, Stefani F, Rinaldo C, Pisciottani A (2019). ESCRT-III is necessary for the integrity of the nuclear envelope in micronuclei but is aberrant at ruptured micronuclear envelopes generating damage. Oncogenesis.

[78] Xia Y, Ivanovska IL, Zhu K, Smith L, Irianto J, Pfeifer CR (2018). Nuclear rupture at sites of high curvature compromises retention of DNA repair factors. J. Cell Biol..

[79] Smith LR, Irianto J, Xia Y, Pfeifer CR, Discher DE (2019). Constricted migration modulates stem cell differentiation. Mol. Biol. Cell..

[80] Bakhoum SF, Ngo B, Laughney AM, Cavallo JA, Murphy CJ, Ly P (2018). Chromosomal instability drives metastasis through a cytosolic DNA response. Nature.

[81] Kim, T., Hwang, W., Lee, H. & Kamm, R. D. Computational analysis of viscoelastic properties of crosslinked actin networks. *PLoS Comput Biol.* **5**, e1000439 (2009). 10.1371/journal.pcbi.1000439 PMC2703781 19609348

[82] Feric M, Brangwynne CP (2013). A nuclear F-actin scaffold stabilizes ribonucleoprotein droplets against gravity in large cells. Nat. Cell Biol..

[83] Le HQ, Ghatak S, Yeung CYC, Tellkamp F, Günschmann C, Dieterich C (2016). Mechanical regulation of transcription controls Polycomb-mediated gene silencing during lineage commitment. Nat. Cell Biol..

[84] Ungricht R, Kutay U (2017). Mechanisms and functions of nuclear envelope remodelling. Nat. Rev. Mol. Cell Biol..

[85] Heo SJ, Han WM, Szczesny SE, Cosgrove BD, Elliott DM, Lee DA (2016). Mechanically induced chromatin condensation requires cellular contractility in mesenchymal stem cells. Biophys. J..

[86] Seong J, Tajik A, Sun J, Guan JL, Humphries MJ, Craig SE (2013). Distinct biophysical mechanisms of focal adhesion kinase mechanoactivation by different extracellular matrix proteins. Proc. Natl Acad. Sci. USA.

[87] Tajik A, Zhang Y, Wei F, Sun J, Jia Q, Zhou W (2016). Transcription upregulation via force-induced direct stretching of chromatin. Nat. Mater..

[88] Price BD, D’Andrea AD (2013). Chromatin remodeling at DNA double-strand breaks. Cell.

[89] Bancaud A, Huet S, Daigle N, Mozziconacci J, Beaudouin J, Ellenberg J (2009). Molecular crowding affects diffusion and binding of nuclear proteins in heterochromatin and reveals the fractal organization of chromatin. EMBO J..

[90] Mao Y, Zhang N, Xu J, Ding Z, Zong R, Liu Z (2012). Significance of heterogeneous Twist2 expression in human breast cancers. PLoS ONE.

[91] Fang X, Cai Y, Liu J, Wang Z, Wu Q, Zhang Z (2011). Twist2 contributes to breast cancer progression by promoting an epithelial-mesenchymal transition and cancer stem-like cell self-renewal. Oncogene.

[92] Padmanaban V, Krol I, Suhail Y, Szczerba BM, Aceto N, Bader JS (2019). E-cadherin is required for metastasis in multiple models of breast cancer. Nature.

[93] Keller L, Pantel K (2019). Unravelling tumour heterogeneity by single-cell profiling of circulating tumour cells. Nat. Rev. Cancer.

[94] Massagué J, Obenauf AC (2016). Metastatic colonization by circulating tumour cells. Nature.

